# Impact of respiratory muscle-stretching exercise on chest expansion and shoulder mobility post-thoracotomy: a randomized controlled trial

**DOI:** 10.1186/s13019-025-03760-9

**Published:** 2025-12-02

**Authors:** Saikaew Chuachan, Rerknapat Jitmana, Umpira Promsri, Pattiyaporn Thongdaeng, Nimit Kosura, Voravit Chittithavorn

**Affiliations:** 1 Department of Physical Therapy, Faculty of Medicine, Prince of Songkla University, Hat Yai, 90110 Songkhla Thailand; 2 Faculty of Physical Therapy, Dhurakij Pundit University, Lak Si, Bangkok, 10210 Thailand; 3 Physical Therapy Unit, Songklanagarind Hospital, Hat Yai, 90110 Songkhla Thailand; 4 Department of Surgery, Faculty of Medicine, Prince of Songkla University, Hat Yai, 90110 Songkhla Thailand

**Keywords:** Thoracotomy, Respiratory muscle-stretching exercises, Deep breathing, Chest wall expansion, Shoulder mobility

## Abstract

**Background:**

Thoracotomy often causes respiratory muscle injury, reduced chest wall expansion, decreased lung volume, and limited shoulder range of motion (ROM). Respiratory muscle-stretching exercises (RMSE) have been proposed to enhance chest expansion and facilitate recovery. This aimed to compare the effects of RMSE combined with conventional physical therapy versus conventional therapy alone in patients undergoing elective thoracotomy.

**Methods:**

A single-center randomized controlled trial was conducted at Songklanagarind Hospital, Thailand, between August 2013 and December 2019. Twenty-eight patients scheduled for elective thoracotomy were recruited, and 23 (mean age 47.1 ± 15.2 years; 18 males, 5 females) completed the trials. Participants were randomized to an intervention group (RMSE plus conventional therapy, *n* = 12) or a control group (conventional therapy only, *n* = 11). RMSE consisted of four stretching exercises performed twice daily for eight days. Primary outcomes were middle and lower chest expansion (MCE, LCE), slow vital capacity (SVC), and shoulder flexion and abduction ROM, assessed preoperatively, on postoperative day 2, and day 8.

**Results:**

Both groups demonstrated significant within-group improvement between days 2 and 8. In the intervention group, MCE increased by 1.46 cm (95%CI: -2.5 to -0.87), LCE by 1.75 cm (95%CI: -2.42 to -1.08), SVC by 438 mL (95%CI: -761 to -115), shoulder flexion by 48.3°, and abduction by 38.8° (95%CI: -57.2 to -20.4). Similar improvements were observed in the control group: MCE 1.18 cm (95%CI: -1.76 to -0.61), LCE 1.27 cm (95%CI: -1.91 to -0.65), SVC 347 mL (95%CI: -690 to -4), shoulder flexion 35.4° (95%CI: -50.6 to -20.2), and abduction 35.7° (95%CI: -54.0 to -17.4). Between-group comparisons showed no significant differences for any outcome (all Group x Time, *p*-value > 0.05).

**Conclusions:**

Respiratory muscle-stretching exercise combined with conventional therapy facilitated significant recovery in chest wall mobility, lung volume, and shoulder motion after thoracotomy. However, these benefits were not significantly greater than those achieved with conventional therapy alone. Clinically, Respiratory muscle-stretching exercise is feasible, safe, and may be considered as an adjunct to standard care.

**Trial registration:**

TCTR20140224004.

## Background

Thoracotomy is a common surgical approach performed for the management of pulmonary and mediastinal diseases [1]. While necessary for treating life-threatening conditions, it is associated with substantial postoperative complications [2, 3]. Injury and inflammation of respiratory and chest wall muscles often occur, contributing to reduced lung expansion, impaired pulmonary compliance, and restricted shoulder and trunk mobility [4, 5]. Previous reports have shown declines of up to 55% in vital capacity (VC) and approximately 30% in functional residual capacity (FRC) following thoracotomy [6, 7]. These physiological impairments, compounded by pain and muscle guarding, not only delay functional recovery but also increase the risk of pulmonary complications and limit activities of daily living [7–9]. Conventional chest physical therapy, including deep-breathing exercises and range-of-motion (ROM) training, is widely recommended for postoperative [10–12]. Respiratory muscle-stretching exercises (RMSE) are specifically designed to elongate shortened thoracic muscles, thereby improving chest wall mobility and enhancing lung volume. RMSE have shown beneficial effects in patients with chronic obstructive pulmonary disease (COPD) [13, 14], and preliminary evidence suggests similar benefits after cardiac or thoracic surgery [16–18]. Aida et al. reported that chest wall stretching promoted greater thoracic expansion than deep breathing after cardiac surgery [15], while Reeve et al. demonstrated that shoulder-mobility training improved ROM, reduced pain, and enhanced quality of life following thoracic surgery [17]. However, most available studies have examined non-pulmonary surgical populations, leaving a gap in evidence regarding the effectiveness of RMSE in patients undergoing pulmonary thoracotomy [11, 14, 16–21]. RMSE may improve thoracic compliance, facilitate lung expansion, reduce the risk of postoperative pulmonary complications, and restore shoulder function. The exercises are low-cost, easy to perform, and can be integrated into rehabilitation protocols. Nonetheless, RMSE require active patient participation, may cause discomfort due to postoperative pain, and lack standardized application protocols across clinical settings, which may limit reproducibility [14–18]. Despite the established role of conventional physiotherapy, its capacity to fully restore chest wall flexibility remains limited [10, 22, 23]. Evidence on RMSE in thoracotomy patients is scarce, heterogeneous, and often lacks rigorous design or standardized outcome measures. Recent reviews emphasize the need for targeted interventions that address both pulmonary function and musculoskeletal limitations after thoracic surgery [19–22]. This study was therefore designed to fill this knowledge gap by directly comparing RMSE with conventional therapy in pulmonary thoracotomy patients. Clarifying the effects of RMSE will provide valuable evidence for clinicians and rehabilitation specialists seeking to optimize recovery strategies, guide patient education, and improve quality of life following thoracic surgery. For researchers, this study contributes to the growing body of literature on postoperative rehabilitation techniques, while patients may benefit from a safe, accessible, and potentially more effective alternative to conventional therapy. We hypothesized that RMSE would lead to greater improvements in chest wall expansion, lung volume, and shoulder ROM compared with conventional chest physical therapy in patients following thoracotomy.

## Methods

### Study design and setting

This prospective, single-center, randomized controlled trial (RCT) was conducted at Songklanagarind Hospital, Hatyai, Songkhla, Thailand, from August 2013 to December 2015. Ethical approval was obtained from the Human Research Ethics Committee of Prince of Songkla University (EC 56–404-11-2), and the trial was registered with the Thai Clinical Trials Registry (TCTR20140224004).

**Fig. 1:**
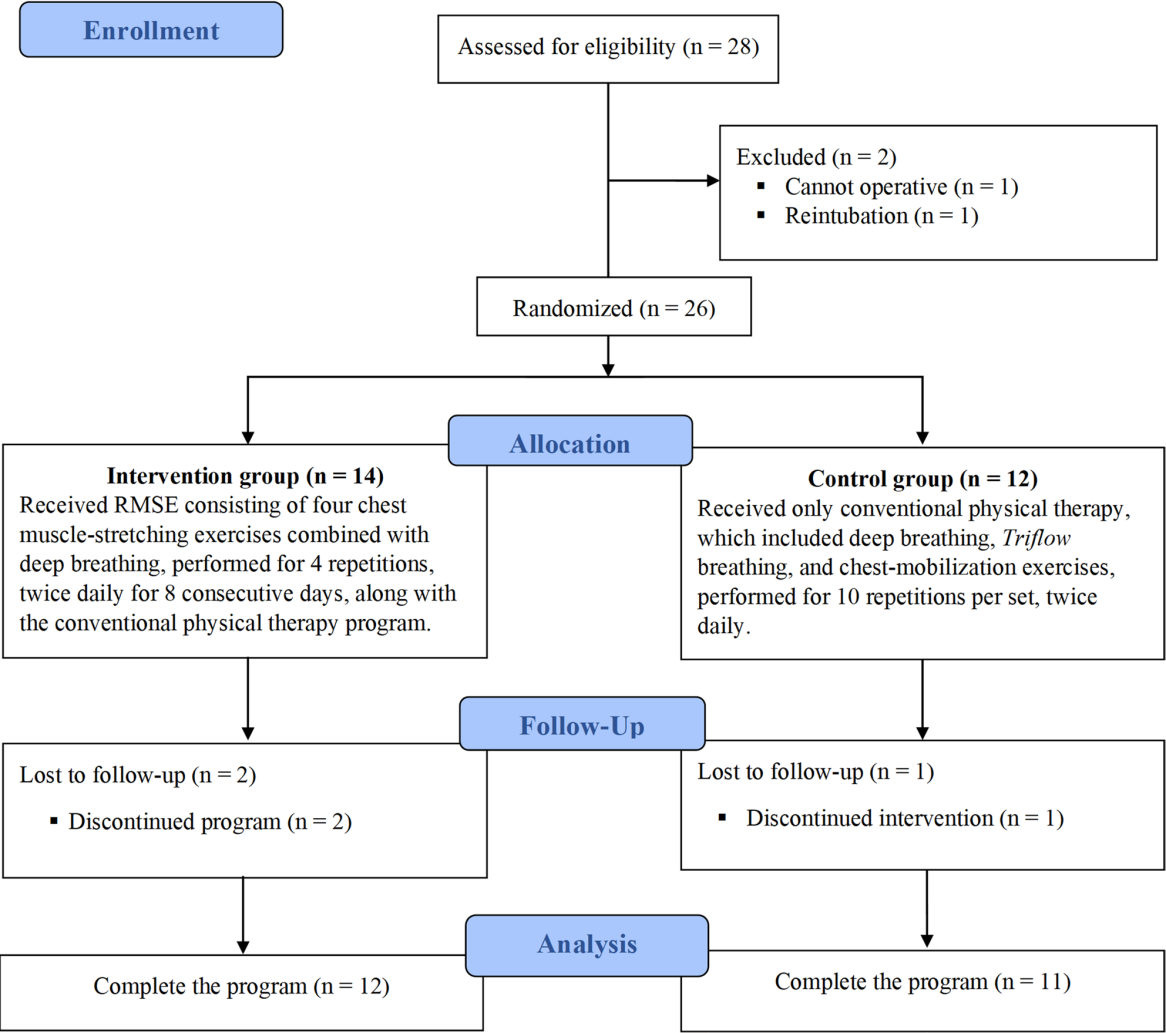
Study flow diagram

### Participants

Patients aged 18–75 years who underwent thoracotomy, were fully conscious, and able to cooperate with postoperative training were eligible for inclusion. Comorbidities were defined as documented chronic conditions, including hypertension, diabetes mellitus, and chronic obstructive pulmonary disease. Patients were excluded if they required mechanical ventilation for more than 72 h postoperatively or experienced respiratory distress requiring re-intubation, underwent pneumonectomy, were unable to maintain posture in upright position, or presented with unstable clinical parameters such as heart rate exceeding 120 beats per minute, respiratory rate over 30 breaths per minute, oxygen saturation below 95%, or systolic blood pressure below 90 or above 140 mmHg. All participants were diagnosed and evaluated by a board-certified thoracic surgeon with over ten years of experience, and written informed consent was obtained before enrollment.

### Randomization and allocation

Participants were randomly assigned to the intervention or control group using block randomization with a block size of four, generated via a computer algorithm. Allocation was concealed using sealed opaque envelopes prepared by an independent researcher who did not participate in outcome assessment or intervention delivery.

### Blinding

Outcome assessors were blinded to group allocation to minimize bias. The independent reviewer supervising the RMSE did not participate in outcome measurements. Although participants were aware of the type of intervention they received, they were instructed not to disclose details to the assessors.

### Interventions

**Fig. 2:**
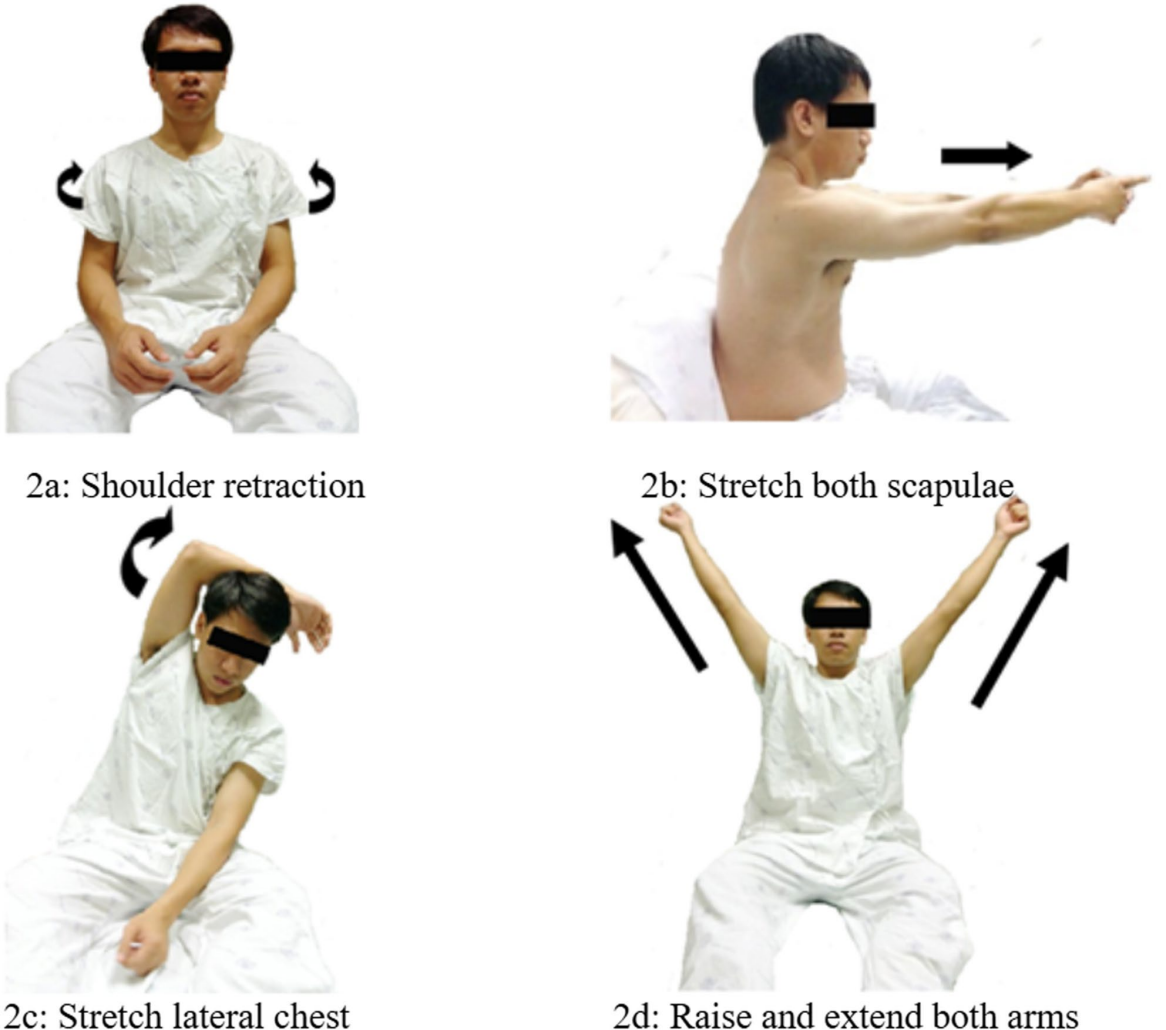
Respiratory muscle-stretching exercises (RMSE)

The intervention group received respiratory muscle stretching exercise (RMSE) in combination with conventional chest physical therapy. RMSE was performed in a 90° upright sitting position and adapted from respiratory muscle-stretch gymnastics. The program included four distinct chest muscle stretches, each performed under mild tension with three deep breaths per stretch, repeated four times. The full sequence was performed twice daily for eight consecutive days. Real-time figures of posture and hand placement are shown in Fig. 2.

All participants received conventional chest physical therapy as standard postoperative care, serving as the control for RMSE efficacy. The conventional therapy protocol included deep-breathing exercises performed in a 90° upright sitting position, with ten repetitions per set, twice daily. *Triflow* breathing exercises involved full exhalation followed by slow inhalation through the device to raise the ball and maintain it for three seconds, performed twice daily with ten repetitions per set. Chest-mobilization exercises included two basic movement patterns combined with deep inhalation, performed in a seated position, with ten repetitions per exercise and two sets per day.

### Outcome measures

**Fig. 3:**
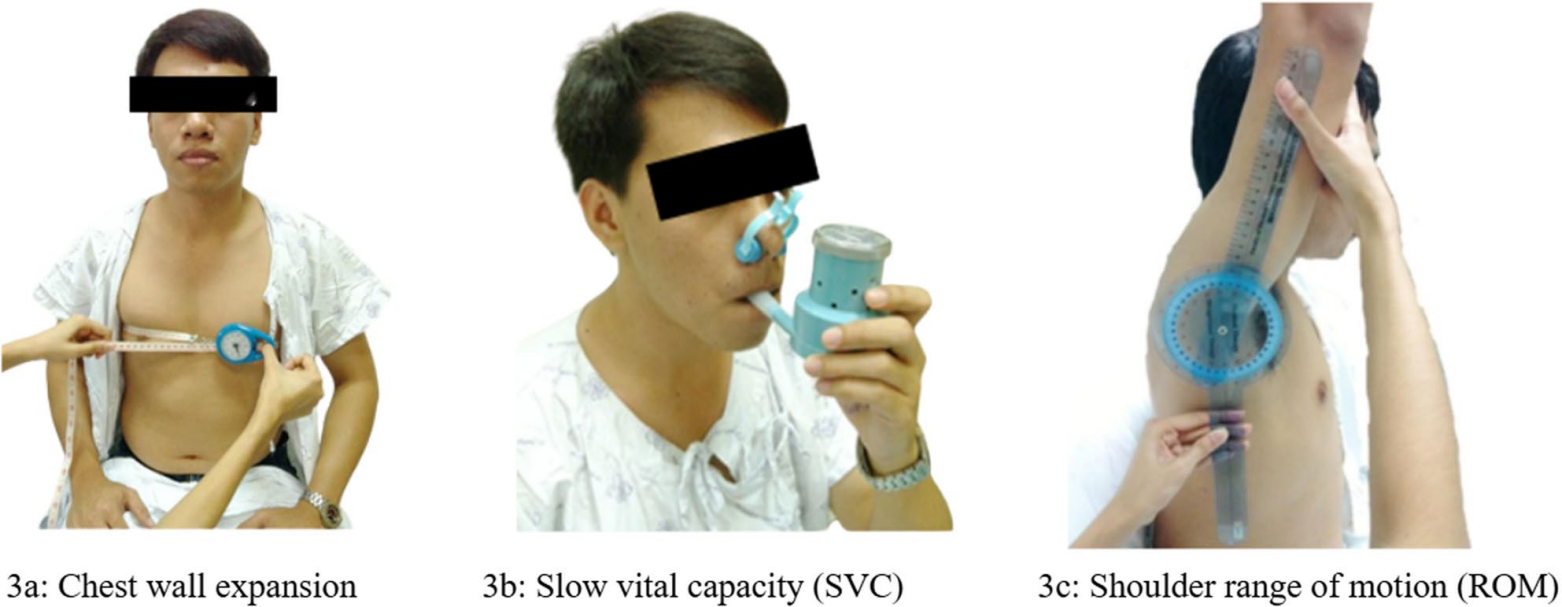
Outcomes measurements

Assessments were conducted preoperatively, daily for eight days postoperatively, and at specific postoperative time points (SVC: days 2 and 8). Vital signs, including heart rate, respiratory rate, blood pressure, and oxygen saturation, were measured using hospital-grade monitors. Pain intensity was evaluated using the Visual Rating Scale, a numerical scale from 0 to 10 with an ICC of 0.91. Dyspnea was assessed using the Modified Borg Scale, with scores ranging from 0 to 10. Shoulder range of motion (ROM) for active abduction and flexion was measured with a goniometer, with the best value of three trials recorded [24]. Lung capacity was assessed by slow vital capacity (SVC) using a spirometer (Riester Spirotest 2650), with the best of three trials recorded [25]. Chest wall expansion was measured at the middle (xiphoid) and lower (8th rib) levels using a tape measure [26], with the best value of three trials recorded (Fig. 3).

### Sample size calculation

Based on a previous study by Minoguchi et al. (2002) [21] evaluating the effect of respiratory muscle stretching exercise on chest expansion post-thoracotomy, the required sample size per group was calculated to achieve 80% power at a 0.05 significance level. Accounting for a 20% dropout rate, 10 participants per group were enrolled, resulting in a total of 20 participants.

### Statistical analysis

Statistical analyses were performed using R software (version 4.3.2). Descriptive statistics were used to summarize baseline demographic and clinical characteristics. Continuous variables were expressed as mean ± standard deviation (SD) or estimated marginal means with 95% confidence intervals (CI), and categorical variables as frequency and percentage. Within-group changes over time (preoperative, postoperative day 2, and day 8) were analyzed using paired t-tests. Between-group differences and the effect of group × time interaction were assessed using generalized linear mixed-effects models with random intercepts to account for repeated measures. Estimated marginal means with 95% CI were reported. The homogeneity of baseline characteristics was examined using Levene’s test.

## Results

A total of 29 patients scheduled for elective thoracotomy were screened, and 25 met the eligibility criteria and completed the trial (12 in the intervention group and 11 in the control group). The flow of the study was presented in Fig. 1.

### Baseline characteristics

Baseline demographic and clinical characteristics were comparable between groups (Table 1). The mean age was 46.1 ± 16.4 years in the intervention group and 48.2 ± 14.5 years in the control group. Most participants were male (83.3% vs. 72.7%). There were no significant between-group differences in body mass index, chest tube duration, or hospital stay. Medical diagnoses included lung cancer, aspergilloma, empyema thoracis, pneumothorax, and tumor. Levene’s test confirmed homogeneity of variance for baseline variables (*p*-value > 0.05).

**Table 1:** Baseline characteristics of participants

Variables	Intervention group (*n* = 12)	Control group (*n* = 11)	*p*-value
Age (years; mean ± SD)	46.08 ± 16.43	48.18 ± 14.53	0.74
Sex, n (%)	10 (83.33%)	8 (72.72%)	0.64^1^
BMI (kg/m^2^)	22.67 ± 2.95	20.34 ± 2.93	0.072
Duration of chest tube placement (days), mean ± SD	5.58 ± 2.27	5.18 ± 3.45	0.74
Length of hospital stay (days) mean ± SD	12.16 ± 3.99	13.45 ± 7.03	0.60
History of alcohol consumption, n (%)	7 (58.33)	5 (45.45)	0.84^2^
History of smoking, n (%)	9 (75)	7 (63.63)	0.66^2^
Co-morbidity, n (%)	2 (16.66)	4 (36.36)	0.37^2^
Medical diagnosis, n (%)			
- Aspergiloma	0(0)	4 (36.36)	
- Lung cancer	10 (83.33)	3 (27.27)	
- Empyema thorasis	1 (8.33)	2 (18.18)	
- Pneumothorax	1(8.33)	1 (9.09)	
- Tumor	0 (0)	1 (9.09)	

^1^Values represent number (%) of males; the remaining participants were females

^2^Values represent number (%) of participants with the condition (alcohol consumption, smoking, or co-morbidity); the remaining participants did not have the condition

^3^*p*-values for surgical region represent separate comparisons for Upper, Middle, and Lower regions

M, male; F, female; n, number; SD, standard deviation; BMI, body mass index

### Chest wall expansion

Estimated marginal means from the linear mixed-effects model (Table 3) showed a reduction in chest wall expansion on postoperative day 2 compared with preoperative values in both groups. From day 2 to day 8, significant within-group improvements were observed (Table 2). In the intervention group, middle chest expansion (MCE) increased by 1.46 cm (*p*-value < 0.001) and lower chest expansion (LCE) by 1.75 cm (*p*-value < 0.001). Similar improvements were seen in the control group (MCE + 1.18 cm, *p* < 0.001; LCE + 1.27 cm, *p*-value = 0.003). However, no significant group × time interaction was detected.

**Table 2:** Estimated marginal means (95% CI) of outcomes by group and time

Group	Pre-operative					Postoperative Day 2 (D2)					Postoperative Day 8 (D8)				
	MCE (cm)	LCE (cm)	SVC (mL)	Sh.F (°)	Sh.Abd (°)	MCE (cm)	LCE (cm)	SVC (mL)	Sh.F (°)	Sh.Abd (°)	MCE (cm)	LCE (cm)	SVC (mL)	Sh.F (°)	Sh.Abd (°)
Intervention (*n* = 12)	3.71(3.10–4.32)	4.50(3.72–5.28)	1758(1478–2039)	173(161–185)	173(158–188)	2.00(3.19–2.61)	2.25(1.47–3.03)	850(569–1131)	110(98–122)	125(111–140)	3.46(2.85–4.07)	4.00(3.22–4.78)	1288(1007–1568)	158(146–170)	164(149–179)
Control (*n* = 11)	3.64(3.00–4.27	4.41(3.59–5.23)	1700(1407–1993)	177(165–190)	178(163–193)	1.73(1.09–2.36)	2.14(1.32–2.96)	782(489–1075)	125(113–138)	133(118–148)	2.91(2.27–3.54)	3.41(2.59–4.23)	1129(836–1422)	160(148–173)	169(153–184)
*p* - value	0.869	0.872	0.733	0.620	0.620	0.534	0.840	0.736	0.083	0.476	0.214	0.298	0.435	0.780	0.668

Values are estimated marginal means with 95% CI from linear mixed-effects models with random intercepts. Group × Time interaction was not statistically significant for any outcome. MCE, middle chest wall expansion; LCE, lower chest wall expansion; SVC, slow vital capacity; Sh.F, shoulder flexion; Sh.Abd, shoulder abduction; cm, centimeter; mL, milliliter; D2, day 2; D8, day 8

**Table 3:** Within-group change over time

Outcome	Group	Contrast	Mean diff (95%CI)	*p*-value
	Intervention	D0-D2	1.71 (1.17–2.25)	< 0.001
		D0-D8	0.25 (−0.37–0.87)	0.629
MCE (cm)		D2-D8	−1.46 (−2.05 to −0.87)	< 0.001
	Control	D0-D2	1.91 (1.34–2.49)	< 0.001
		D0-D8	0.73 (0.05–1.40)	0.036
		D2-D8	−1.18 (−1.76 to −0.61)	0.0004
	Intervention	D0-D2	2.25 (1.56–2.94)	< 0.001
		D0-D8	0.50 (−0.32 −1.32)	0.321
LCE (cm)		D2-D8	−1.75(−2.42 to −1.08)	< 0.001
	Control	D0-D2	2.27 (1.55–3.00)	< 0.001
		D0-D8	1.00 (0.15–1.86)	0.021
		D2-D8	−1.27 (−1.91 to −0.65)	0.003
	Intervention	D0-D2	908 (634–1182)	< 0.001
		D0-D8	471 (169–773)	0.004
SVC (mL)		D2-D8	−438 (−761 to −115)	0.007
	Control	D0-D2	918 (629–1207)	< 0.001
		D0-D8	571 (286–856)	0.001
		D2-D8	−347 (−690 to −4)	0.049
	Intervention	D0-D2	63.2 (47.7–78.7)	< 0.001
		D0-D8	14.9 (−2.1 −31.9)	0.126
Sh.F (°)		D2-D8	−48.3 (−63.1 to −33.5)	< 0.001
	Control	D0-D2	52.2 (36.6–67.8)	< 0.001
		D0-D8	16.8 (−1.3-34.9)	0.091
		D2-D8	−35.4 (−50.6 to −20.2)	0.0001
	Intervention	D0-D2	47.6 (29.2–66.0)	< 0.001
		D0-D8	8.8 (−10.6 −28.3)	0.604
Sh.Abd (°)		D2-D8	−38.8 (−57.2 to −20.4)	0.0004
	Control	D0-D2	45.3 (26.9–63.7)	0.0001
		D0-D8	9.6 (−10.1–29.2)	0.583
		D2-D8	−35.7 (−54.0 to −17.4)	0.0016

MCE, middle chest wall expansion; LCE, lower chest wall expansion; SVC, slow vital capacity; Sh.F, shoulder flexion; Sh.Abd, shoulder abduction; Pre, preoperative; D2, postoperative day 2; D8, postoperative day 8

### Lung function

Slow vital capacity (SVC) declined markedly at day 2 and improved significantly by day 8 in both groups. Within-group increases were 438 mL in the intervention group (*p*-value = 0.007) and 347 mL in the control group (*p*-value = 0.049). Between-group differences were not statistically significant (*p*-value = 0.736, Table 3).

### Shoulder range of motion

Shoulder flexion and abduction decreased substantially at day 2 in both groups. By day 8, recovery was evident, with flexion improving by 48.3° (*p*-value < 0.001) and abduction by 38.8° (*p*-value < 0.001) in the intervention group. The control group showed similar improvements, with flexion increasing by 35.4° (*p*-value < 0.001) and abduction by 35.7° (*p*-value = 0.002). No significant between-group differences were observed (all group × time *p* > 0.05).

## Discussion

This randomized controlled trial examined the effects of respiratory muscle-stretching exercises (RMSE) in addition to conventional chest physiotherapy compared with conventional therapy alone in patients undergoing thoracotomy. The main finding was that both groups demonstrated significant recovery in chest expansion, lung function, and shoulder mobility between postoperative days 2 and 8, but no significant differences were observed between groups.

Our results are consistent with prior research in patients with COPD and thoracic surgery, which showed that chest wall stretching and deep breathing improve thoracic expansion and lung function [14, 16, 18, 21, 27]. Similar to Minoguchi et al., [21] we observed an increase in middle and lower chest expansion of approximately 1–1.5 cm. However, unlike studies that reported superior benefits of stretching over standard care [16, 18], our trial did not demonstrate between-group differences due to postoperative conditions. Moreover, this discrepancy may relate to the additional exercises encouraged by ward staff, which may have diluted group contrasts.

After thoracotomy, trauma and inflammation can occur in the chest wall muscles as well as in the muscles responsible for shoulder and trunk movements. This reduces the muscle function and restricts lung expansion [4, 5]. Previous studies have reported that thoracotomy can lead to a 55% reduction in VC and a 30% reduction in FRC [6, 7]. In this study, most participants underwent lobectomy (16 patients, eight in the intervention group and eight in the control group), whereas four patients underwent decortication (two in each group). The postoperative assessment of SVC revealed a trend toward improvement. However, no statistically significant differences were observed between groups. Interestingly, the control group exhibited slightly greater improvements in lung volume than the intervention group. This finding may be explained by the location of the resected lung lobes. In the intervention group, four patients underwent upper lobe resection, three lower lobe resections, and three middle lobe resections. In contrast, the control group included six patients who underwent upper lobe resection and one with lower lobe resection. Previous findings suggest that the upper lobes tend to expand more readily and recover faster than the lower lobes [27, 28]. Therefore, the higher number of upper lobe resections in the control group might have contributed to the greater improvement in lung expansion.

Shoulder mobility, particularly flexion and abduction, is frequently impaired during the early postoperative period, affecting overall upper limb and trunk functions [5, 6]. After 8 days of chest wall stretching, deep breathing, and standard postoperative physical therapy, both the intervention and control groups showed improved shoulder flexion and abduction, with no significant difference between the groups.

Both RMSE and conventional therapy rely on active inspiration and thoracic mobilization. In the early postoperative period, improvements may be driven more by natural recovery [29], pain reduction, and repeated practice of deep breathing than by the specific stretching technique. The small effect sizes and wide confidence intervals observed in this study suggest that RMSE may not confer clinically meaningful benefits beyond standard therapy within the short postoperative timeframe.

No serious adverse events were reported. Mild discomfort at the incision site and transient muscle soreness occurred in two participants in the intervention group; these were managed conservatively with rest and reassurance, and did not lead to withdrawal.

Several unmeasured variables may have influenced outcomes, including differences in analgesic use, pain characteristics, age-related recovery capacity, and adherence to prescribed exercises. In addition, informal exercises promoted by nursing staff (e.g., upper limb movements and Triflow breathing) may have overlapped with the intervention, reducing contrast between groups.

### Strengths and limitations

The study’s strengths include its randomized controlled design, blinded outcome assessment, and clinically relevant outcomes. However, limitations include the small sample size, limited generalizability due to single-center design, variability in surgical procedures (lobectomy vs. decortication), and patient discomfort in the early postoperative days, which reduced adherence. Real-time challenges, such as postoperative pain, fatigue, and variable cooperation, constrained the consistency of data collection and may have influenced results. Wide confidence intervals further highlight the uncertainty in effect estimates.

### Clinical implications

RMSE is safe and feasible after thoracotomy. Although not superior to conventional therapy, it may support patient motivation and adherence to rehabilitation. Future studies with larger samples are required to determine whether its use translates into clinically meaningful long-term benefits.

## Conclusions

In patients undergoing thoracotomy, RMSE combined with conventional therapy facilitated recovery of chest wall expansion, lung function, and shoulder mobility. However, the effects were comparable to conventional therapy alone. These findings suggest that while RMSE is a safe and practical addition, its incremental benefit remains uncertain and requires confirmation in larger studies.

## Data Availability

No datasets were generated or analysed during the current study.

## Abbreviations

RMSE: Respiratory muscle-stretching exercise
SVC: Slow vital capacity
MCE: Middle chest expansion
LCE: Lower chest expansion
VC: Vital capacity
FRC: Functional residual capacity
COPD: Chronic obstructive pulmonary disease
